# Genetic and Clinical Features of Schimke Immuno-Osseous Dysplasia: Single-Centre Retrospective Study of 21 Unrelated Paediatric Patients over a Period of 20 Years

**DOI:** 10.3390/ijms26041744

**Published:** 2025-02-18

**Authors:** Anastasiia Milovanova, Petr Ananin, Tatiana Vashurina, Olga Zrobok, Svetlana Dmitrienko, Alla Ryaposova, Elena Tsygina, Alexander Pushkov, Ilya Zhanin, Daria Chudakova, Aliy Asanov, Olga Shchagina, Aleksander Polyakov, Andrey Fisenko, Kirill Savostyanov, Alexey Tsygin

**Affiliations:** 1 Federal State Autonomous Institution “National Medical Research Center of Children’s Health” of the Ministry of Health of the Russian Federation, 119991 Moscow, Russia; 2 Department of Medical Genetics, N.V. Sklifosovsky Institute of Clinical Medicine, I.M. Sechenov First Moscow State Medical University, 115552 Moscow, Russia; 3 Research Centre for Medical Genetics, 115522 Moscow, Russia

**Keywords:** Schimke immuno-osseous dysplasia (SIOD), hereditary nephrotic syndrome, steroid-resistant nephrotic syndrome (SRNS), genetics of rare disease, ultra-rare hereditary disease, *SMARCAL1*

## Abstract

Schimke immuno-osseous dysplasia (SIOD) is a hereditary autosomal-recessive multi-system disorder with early mortality. It has variable clinical presentations, mainly characterised by disproportional short stature, steroid-resistant nephrotic syndrome, spondyloepiphyseal dysplasia, and T-cell immunodeficiency. In the majority of cases, SIOD is caused by pathogenic sequence variants (PSVs) in the *SMARCAL1* gene that encodes protein involved in chromatin remodelling. SIOD is an ultra-rare condition, with an incidence of ~1 per 1–3 million live births; data on its genetic and clinical features are scarce. We conducted a retrospective study of 21 paediatric patients with SIOD diagnosed in our centre during the years 2003–2023. The most common extra-renal clinical features were short stature, osseous dysplasia, multiple stigmas, and leukopenia. Proteinuria of varying severity was observed in 16 cases. The five-year overall survival rate (OS) was 89% (95% CI 77–100%), and the ten-year OS was 10%. Next-generation sequencing (NGS) revealed the following PSVs in *SMARCAL1* in 19 patients: *c.355_500del*, *c.2542G>T*, *c.2290C>T*, *c.2562del*, *c.2533_2534del*, *c.1582A>C*, *c.1933C>T*, *c.1010T>C*, *c.1736C>T*, *c.2070dup*, *c.2551A>T*, *c.2149_2150dup*, *c.939delC*, and *c.1451T>A*; the most common was *c.2542G>T*, resulting in premature translation termination (p.E848*), and it was found in 14 patients either in a homozygous (four patients) or compound-heterozygous (10 patients) state. According to microsatellite analysis, it is a “founder mutation” in Russia.

## 1. Introduction

Schimke immuno-osseous dysplasia (SIOD, MIM #242900), a condition first described by R. Schimke in 1971 [1], is an extremely rare hereditary autosomal-recessive disorder, characterised by a combination of growth retardation (disproportional short stature), steroid-resistant nephrotic syndrome (SRNS), spondyloepiphyseal dysplasia, and a T-cell immune defect [2]. It can also be accompanied by episodic thrombocytopenia, abnormal skin pigmentation, a high risk of acute cerebrovascular accidents, and dental disorders. Many SIOD patients have intrauterine growth retardation, and they are usually intellectually intact [3]. Considering the phenotypic manifestations of the disease, the diagnosis of SIOD can be made after a physical examination, followed by a series of laboratory tests (general urinalysis, clinical blood test, biochemical blood test). The incidence of SIOD is 1:1–3 million live births. At the same time, among patients with genetically determined nephrotic syndrome (NS), SIOD occurs in 2.4–9.4% of cases [4,5,6].

SIOD is an extremely unfavourable condition for life, the early mortality of which is caused mostly by infectious complications of agranulocytosis and acute cerebrovascular accidents, besides the most common complications of end-stage renal disease (ESRD) [4]. To date, despite the development of renal replacement therapies (RRT) and all the advances in critical care, the average age of death of these patients is 11 years, and the treatment tactics are still limited to syndrome-based treatment and RRT. Nevertheless, whereas some patients develop a severe form of the disease with in utero onset, individuals with mild cases of SIOD might survive into adulthood, and cases of survival for more than 20 years have been reported [5,7].

Commonly, SIOD is caused by bi-allelic PSVs in the *SMARCAL1* gene [Gene ID: 50485; NG_009771.1] [8]. It encodes one of the nuclear proteins, SWI/SNF-related matrix-associated actin-dependent regulator of chromatin subfamily A-like protein (SMARCAL1), also known as HepA-related protein (HARP). SMARCAL1 belongs to the SNF2 (sucrose non-fermenting type 2) motor protein family and is involved in chromatin remodelling, DNA replication, and repair [9,10]. Its major role is genomic stability maintenance through DNA double-strand break repair by homologous recombination; in line with this, an association of PSVs in *SMARCAL1* with a chromosomal breakage phenotype has been reported in some cases of severe SIOD [11]. Given the role of genomic instability in cancer, it is not surprising that SIOD is also associated with several malignancies [12].

Some patients with SIOD, however, do not harbour apparent PSVs in *SMARCAL1.* This might be due to PSVs located in deep intron regions of *SMARCAL1* [12], the impact of epigenetic and environmental factors, and the possibility of the existence of other genes involved in the pathogenesis of SIOD (oligogenic inheritance) [13]. Notably, according to a previous study report, patients with SIOD but without PSVs in *SMARCAL1* had normal levels of SMARCAL1 mRNA and protein [13].

Furthermore, although PSVs in the *SMARCAL1* gene have been found both in mild [14] and severe [15] SIOD, it is challenging to establish a genotype–phenotype correlation (or its absence) because of the rarity of this disease and, hence, scarcity of the data. Nevertheless, there is a tendency for patients with truncating mutations in *SMARCAL1* to have a more severe form of the disease, whereas missense mutations lead to milder forms. At the same time, multiple studies indicate that patients with the same PSVs in *SMARCAL1*, even siblings, might have different clinical manifestations of SIOD and degrees of severity ([16,17,18], to name but a few). This might be due to the role of SMARCAL1 as a chromatin modifier. Taking into account that changes in chromatin structure might affect a wide array of genes in different loci with a potential impact on SIOD phenotype and that patterns and dynamics of chromatin modification are affected by multiple environmental factors, its plausible to suggest that, as it has been suggested, SIOD phenotype “will reflect the sum of the inter-patient variability in all these processes” [18]. Alternatively, PSVs in other genes, levels of expression of other genes (so-called modifier genes), and overall “individual genomic background” are genetic factors that supposedly might have a role in determining SIOD phenotype.

Given the rarity of SIOD, both genetic and clinical features of this disease (including response to therapy) remain understudied, and any new data are of obvious value. In this work, we address this problem by presenting the results of a retrospective study of 21 unrelated paediatric patients with SIOD over a period of 20 years. To the best of our knowledge, our cohort is the largest single-centre cohort of such patients in the world [19], and this study is the first one providing a detailed characterisation of Russian patients with this ultra-rare condition.

## 2. Results

### 2.1. Clinical Characteristics

In our study, SIOD was diagnosed in 21 unrelated children. All parents denied consanguineous marriage and a family history of kidney disease. The characteristics of the cohort are presented in Table 1.

The majority of children (94%) were born prematurely. The average gestational age at birth was 34 weeks ± 2 weeks. All children were born with low, very low, and extremely low body weight: the average birth weight was 1491 g ± 355 g, SDS −2.03 (−2.18; −1.62). The average height at birth was 41 cm ± 4 cm, SDS −1.49 (−2.11; −0.83 *).

Intrauterine growth retardation was noted in 4/16 (25%), hypotrophy was observed in 5/16 (31%). Only 7/16 (44%) children at birth had anthropometry data corresponding to the gestational age.

The median age of reported proteinuria was 35 (14; 45) months. Among all children, only one (5%) had renal manifestations up to 3 months of age (congenital NS), and two (10%) had renal manifestations between 3 and 12 months of life (infantile NS).

The onset of proteinuria of varying severity (including NS) was noted in 76% of cases (16/21 children), arterial hypertension (AH) at the start of the disease was noted in 10% (2/21 children), with both haematuria and AH detected in two more (4/21; 10%).

At onset, median proteinuria was 1 g/L (0.6 g/L; 2.8 g/L), while oedema was noted in only 2/21 children (10%) and did not demonstrate a significant character; ascites was not observed in any child. Secondary hypothyroidism caused by proteinuria was found in 7/21 children (33%). All children had secondary hypercholesterolemia: 14.66 (10.33; 18.46) mmol/L.

**Figure 1:**
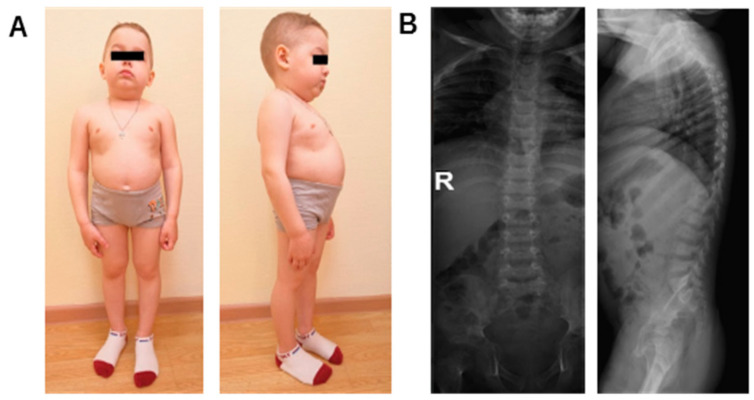
Common clinical features of patients with SIOD in our cohort. (**A**) Six-year-old boy with SIOD: short stature, disproportional short trunk, short neck, low auricles; (**B**) The spine X-ray of a child with SIOD. R—right side. In the lateral projection, the thoracic vertebrae are rounded, the anterior contours are smoothed, and the lumbar vertebrae are flattered. The height of the intervertebral spaces is significantly reduced in the thoracic region throughout and expanded in the lumbar region (due to deformation of the vertebral bodies).

All children included in the cohort had short stature due to a disproportionately short trunk (Figure 1A) and bone anomalies (barrel chest, flattening of the vertebrae, chondrodysplasia, gothic palate) (Figure 1B).

**Figure 2:**
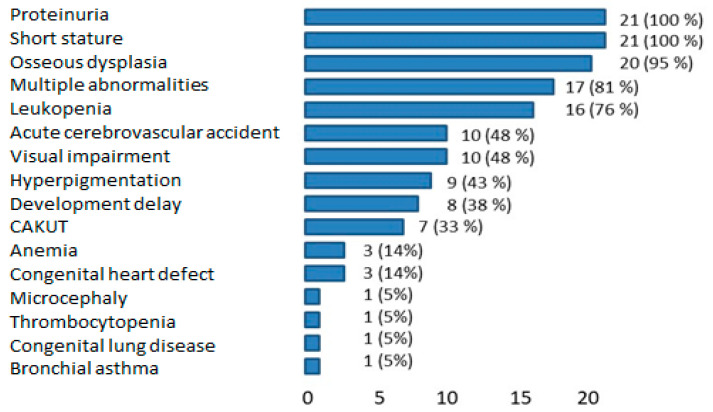
Spectrum of disease manifestations in children with SIOD. CAKUT—congenital anomalies of kidney and urinary tract.

Also, among the extra-renal manifestations, there were leukopenia and T-cell deficiency (CD4+ and CD8+). Lymphopenia was noted in 14/21 children—67%. Only 3/21 (14%) children had neutropenia—patients № 1, 7, and 16. In all children, CD3 positive cells were below reference at 537 (279; 631) per μL, CD4 positive cells were 157 (58; 253) per μL, and CD8 positive cells were 351 (182; 395) per μL. CD19 positive cells had low levels in 5/8 children—in 63%. Multiple “coffee with milk” hyperpigmented spots on the skin and ocular manifestations (photophobia, macular hypoplasia, partial atrophy of the optic nerve, excavation of the optic discs, congenital cataract) were noted in one-half of the patients (10/21 patients, 50%). One-third (8/21) had delayed motor development and/or cognitive deficit before stroke episodes. The majority of patients in the group (17/21, 81%) had more than five mild abnormalities/stigmas (wide nose tip, long filter, thin upper lip, elongated chin, short neck, large dysplastic auricles) (Figure 2, Table 2).

### 2.2. Therapy Outcomes

**Figure 3:**
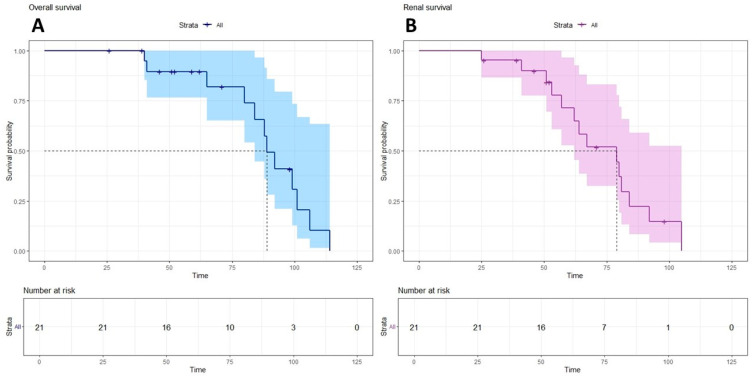
Survival of SIOD patients. (**A**) Overall and (**B**) renal survival. Kaplan–Meier curves.

At the first visit to the clinic, due to unclear phenotypic manifestations, 6/21 (29%) children underwent a kidney biopsy, and focal segmental glomerular sclerosis was revealed in 5/6 (84%) patients (patients № 2, 8, 18, 19, and one child without any pathogenic variants in *SMARCAL1*). Patient № 4 had a pathology of focal global glomerular sclerosis. Nine children (patients № 2, 3, 4, 6, 8, 12, 14, 17, and one child without any pathogenic variants in *SMARCAL1)* (43%) received a course of steroid therapy when proteinuria was initially detected. In five (24%) children (patients № 2, 4, 8, 17, and one child without any pathogenic variants in *SMARCAL1*) without evident SIOD features, we tried treatment with the cacineurin inhibitors cyclosporine A or tacrolimus before molecular genetic tests were performed. In three children, we had to stop this treatment due to its ineffectiveness within 3–6 months (patients № 2, 4, and one child without any pathogenic variants in *SMARCAL1*). In patient № 17, we stopped therapy after one month when we received preliminary data of genetic testing suggesting SIOD. In patient № 8, after 1 month of therapy, partial remission of the NS had been achieved (proteinuria decreased from 3–3.9 g/L to 0.1–0.3 g/L) without a decrease in renal function; the effect persisted throughout the entire course of therapy. However, after 1 year and 6 months, due to increasing creatinine, cyclosporine A was stopped. Three children, patients № 2, № 3, and № 18, received a kidney transplant (№ 2, 3—transplantation from a related donor, № 18—cadaveric kidney transplant); after recovering from surgery and with a functioning graft, all of them died at the ages of 88, 101, and 114 months (7.33 years, 8.42 years, and 9.5 years, correspondingly) of SIOD extra-renal complications. Overall, more than one-half of the children (57%, 12/21 patients) died during the observation period (patients № 1–6, 8, 9, 14, 18, 19, and one child without any pathogenic variants in *SMARCAL1*); three of them had not reached chronic kidney disease (CKD) stage 5 (patients № 1, 4, and 6). The median age of death was 89 months. The five-year overall survival rate was 89% (95% CI 77–100%) (Figure 3), and all children that died were at an age under 10 years.

An additional 6/21 (29%) patients were lost to follow-up (mean age at loss to follow-up was 58 months ± 27 months; 4.83 ± 2.25 years, correspondingly). Three children (14%) are still alive. The median age of the children from the cohort who are currently alive is 31 months ± 14 months (2.58 ± 1.16 years). More than one-half of the children had reduced kidney function to CKD stage 3 by the age of 55 ± 17 months (4.58 ± 1.42 years), patients № 2–6, 8–10, 12–14, 17–19, and one child without any pathogenic variants in *SMARCAL1*, and CKD stage 5 was reached by the age of 62 ± 21 months (5.17 ± 1.75 years) in patients № 2, 3, 5, 8, 9, 12–14, 18, 19, and one child without any pathogenic variants in *SMARCAL1*. The eldest child (patient № 19) achieved stage 5 CKD by the age of 105 months (8.75 years). Three-year renal survival was 95 (95% CI 87–100%), and five-year renal survival was 71 (95% CI 53–97%). Among the causes of death, acute cerebrovascular accidents (6/12; 50% of patients) (patients № 1, 2, 4, 9, 19, and one child without any pathogenic variants in *SMARCAL1*) and sepsis due to immunodeficiency (3/12; 25% of patients) (patients № 8, 14, and 18) were the most frequent; two children (17%) died due to uncontrolled oedema and hydrothorax (patients № 5 and 6), and there were no data on one child’s (8%) cause of death (patient № 3). Growth hormone therapy was attempted in two children (2/21; 10%), patients № 14 and № 19, without any effect.

### 2.3. Genetic Analysis

Genetic analysis of two (2/21; 10%) patients was not performed for technical or ethical (parental refusal) reasons, but for them, SIOD symptoms were also evident. Of the 19 patients for whom genetic analysis was performed, in 17 patients, PSVs in the *SMARCAL1* gene were found in the homozygous or compound-heterozygous state, and in two patients, PSVs were found in the heterozygous state (Table 3). The compound-heterozygosity of PSVs was confirmed in all cases by genetic tests of the patients’ parents.

In the majority (14/19; 73%) of genotyped patients, genetic analysis revealed the PSV *c.2542G>T*, p.E848* in *SMARCAL1*, and in four of patients with p.E848*, it was in the homozygous state (4/14; 29%). Other PSVs in *SMARCAL1* were *c.355_500del*, *c.2290C>T*, *c.2562del*, *c.2533_2534del*, *c.1582A>C*, *c.1933C>T*, *c.1010T>C*, *c.1736C>T*, *c.2070dup*, *c.2551A>T*, and *c.2149_2150dup.*

Microsatellite analysis of microsatellite loci D2S1345, D2S2382, and D2S163, located upstream and downstream from the *SMARCAL1* gene, revealed that haplotype 1-1-5, as determined by markers D2S1345, D2S2382, and D2S163, is likely a founder haplotype that has gradually eroded over time (Table 4, haplotype 1-1-5 highlighted with grey).

The results of microsatellite analysis, including the Fisher criterion calculations for marker alleles with the highest values of the coupling imbalance parameter δ, are summarised in Table 5.

The geographic distribution of *c.2542G>T*, p.E848* was as follows: five children with this variant lived in the Central Federal District (FD) of Russia, three lived in the Volga FD, two lived in the North Caucasus, one lived in Ural FD, one lived in the Southern FD, one lived in Far Eastern FD, and one came from another country (Moldova).

## 3. Discussion

Clinical descriptions of SIOD patients typically include NS without or with mild oedema and a spectrum of extra-renal symptoms affecting the bone, immune, nervous, and vision systems. Isolated cases of congenital heart and kidney defects in SIOD have also been reported [17]. In addition to the common clinical features, multiple specific signs have been described in SIOD patients: triangular face, wide filter, pointed nose tip, hair abnormalities, multiple hyperpigmented spots, and high voice pitch [3].

Taking into account the strikingly specific phenotype (short stature, etc.), the diagnosis of SIOD in most cases can be made clinically before obtaining the results of molecular genetic tests. However, in some patients with SIOD, only NS and short stature are evident during the initial examination, which makes the molecular genetic study mandatory for children with steroid-resistant NS [4]. Alarmingly, as reported in the literature, the use of steroid therapy for children with phenotypic features clearly suggesting SIOD [19] might indicate rather low awareness of the hereditary forms of NS and tactics for SIOD management. In our opinion, patients presenting with disproportional growth failure should be referred for genetic testing to assess the presence of PSVs in *SMARCAL1* to avoid overtreatment with immunosuppressive agents in the case of SIOD.

Here, we summarised clinical observations and genetic features of children with SIOD who, for a period of 20 years, have been observed in the nephrology department of our centre. As aforementioned, this cohort is one of the largest cohorts in the world.

To date, no correlation has been established between certain genetic variants and the severity or spectrum of SIOD manifestations. Due to the fact that the pathogenic variant *c.2542G>T*, p.E848*, first described by Boerkoel in 2002 in another population, was found in most patients in our cohort, it was not feasible to study a genotype–phenotype correlation in our work either. Such a high frequency of variant *c.2542G>T*, p.E848* in our cohort as well as the results of microsatellite analysis identify it as a major causative one and indicate its founder effect in the Russian Federation. Moreover, we found that haplotype 1-1-5, as determined by markers D2S1345, D2S2382, and D2S163, is likely a founder haplotype.

According to the results of the molecular genetic study, only one PSV was found in *SMARCAL1* in a heterozygous state in two patients (patients № 17 and № 18), variants *c.2542G>T*, p.E848* and *c.2459G>A*, p.R820H, respectively. As both these patients were deceased, we could not obtain fresh biological material for further “in depth” molecular-genetic analysis, including analysis of the deep intron regions of the *SMARCAL1* gene or whole-genome sequencing. For the same reasons, we could not study *SMARCAL1* transcripts in patients 17 and 18 to assess if wild-type transcript is detectable in their cells. Thus, we do not exclude the possibility of the presence of PSVs in those regions or other genetic alterations affecting *SMARCAL1*. Previously, it has been suggested that, for such patients, second-tier genome sequencing is the best strategy for molecular diagnostics [20]. Previously, several cases of SIOD caused by PSVs in intronic regions of *SMARCAL1* [21,22] had been described in different populations.

In our study, apart from *c.2542G>T*, the following PSVs in *SMARCAL1* were found: *c.355_500del*, *c.2290C>T*, *c.2562del*, *c.2533_2534del*, *c.1582A>C*, *c.1933C>T*, *c.1010T>C*, *c.1736**C**>T*, *c.2070dup*, *c.2551A>T*, and *c.2149_2150dup*. The diagnostic utility and presence in patients with SIOD have been demonstrated previously for some of these variants (i.e., *c.2290C>T*, *c.1933C>T*, *c.1736C>T*) [8,23].

As for the therapy tactics and outcomes, the observed effect of calcineurin inhibitors in patient № 9 in our cohort can be explained by the so-called podocytoprotective effect, achieved through the intraglomerular haemodynamics, inhibition of synaptopodin degradation, stabilisation of the actin cytoskeleton of podocytes as previously reported [24], and changes in cofilin-1 expression [25]. Furthermore, the literature describes one case of partial remission in a SIOD patient receiving calcineurin inhibitors [26].

Taking into account the rapid decline in renal function while taking potentially nephrotoxic therapy, this tactic cannot be recommended if a hereditary form of NS is suspected, especially SIOD (extremely high risk of a more rapid decline in renal function in children with a potentially unfavourable prognosis and increased risk of infectious complications in children with T-cell deficiency). The late age of proteinuria detection in patients with hereditary NS could be explained by poor diagnostics and the prevalence of patients’ complaints not related to a kidney disorder (low growth, leukopenia, neurological symptoms), forcing diagnostic efforts to be focused on “more actual” problems. In our study, the average age of proteinuria detection in patients with SIOD was 2 years 6 months, median—2 years 11 months.

Previously published data report the overall patient survival rate at age 10 years as 54 ± 10% [27]. More than one-half of the children from our cohort (11/21 children; 52%) died during the observation period; eight of them had required renal replacement therapy. Notably, there was a difference in the renal disease manifestation in the Lipska-Ziętkiewicz et al. cohort and our cohort; in our cohort, the median onset age was 2 years (1.2; 3.8), and in the study by Lipska-Ziętkiewicz et al., the renal disease first manifested at 4.5 years (3.2; 7.7) [27]. Similar to our cohort, the most common PSV reported by Lipska-Ziętkiewicz et al. in their cohort was *c.2542G>T* (p.E848*), found in 10 patients of a total of 34; in all but one case, p.Glu848* was in a compound-heterozygous state with missense mutations. Subsequent genotype–phenotype correlation analysis demonstrated that patients with bi-allelic missense mutations had a benign phenotype compared to those harbouring bi-allelic truncating mutations [27]. Given that, in our study, most of the patients had the *c.2542G>T* (p.E848*) truncating mutation in a homozygous state, it might be one of the explanations why the median onset age was lower in our cohort.

Among the causes of mortality in SIOD patients, acute cerebrovascular accidents are the leading ones. The association of SIOD and vascular features leading to acute cerebrovascular accident was first described by Spranger et al. [28]—four patients had atherosclerotic changes in cerebral vessels. Ehrich et al. [29] described three patients with transient ischemic attacks, but no neuroimaging pathology was detected. In our study, almost half of the patients experienced acute cerebrovascular accidents.

Several groups—Boerkoel et al. [8] in 1998 and, much later, others [30,31,32]—independently reported an observation of features of Moyamoya syndrome (MMS) in magnetic resonance imaging (MRI) in patients with SIOD from different geographical regions. MMS is characterised by a narrowing of the internal carotid arteries’ intracranial segments and the anterior and middle cerebral arteries up to its occlusion. A striking distinctive feature of MMS is the formation of a network of collateral vessels at the base of the brain, which looks like a “haze” on angiograms [33]. The pathophysiological mechanism of the possible link between MMS and SIOD has not been elucidated. The thickening of the tunica intima and media compatible with arteriosclerosis in the aortas of patients with SIOD compared to controls and cerebral arteriosclerosis has been described [34]. Also, a defect in elastogenesis has been proposed as a cause of atherosclerosis when reduced expression of tropoelastin and elastin has been found in foetal umbilical arteries of patients with SIOD [35]. Interestingly, in our study cohort, there were no patients with MMS.

Although hyperlipidaemia can be explained by compensatory increasing liver protein synthesis due to expressed urinary protein losses and hypoalbuminemia, it has been suggested that SMARCAL1 might also modulate cellular lipid metabolism due to its interaction with a well-known regulator of lipoprotein lipase and endothelial lipase, angiopoietin-like 3 (Angptl3) [36]. Furthermore, a role of SMARCAL1 in nephrogenesis has been suggested based on animal and human studies [37,38]. This might explain the high cholesterol levels in all our children—14,66 (10,33; 18,46) mmol/L.

One of the predominant causes of mortality in SIOD patients is multiple infectious complications. Immunodeficiency in SIOD includes such components as cell-mediated T-cell immunodeficiency, combined immunodeficiency with profound lymphopenia, lack of thymic production, deficient IL-7R expression, altered differentiation of plasma cells and immunoglobulin production, and altered NK-cell function and phenotype [17]. In our cohort, lymphopenia was noted in 14/21 children (patients № 1–10, 13, 14, 17, and 19)—67%. Only three children had neutropenia—patients № 1, 7, and 16. In all children, the numbers of T cells and NK cells were below the references. Low levels of B cells were observed in more than half of the patients (in 5/8 children, 63%).

In some clinics, bone marrow transplantation is used as a solution for “the immunodeficiency problem”, but effectiveness comes with great risks of strokes, infections, and graft versus host disease that can increase mortality (survival in some clinics is approximately 54% to the age of 10 years) [19,27,39]. Nevertheless, some encouraging results are described in three cases of sequential αβ haploidentical T cells and B cells, depleted hematopoietic stem cells, and kidney transplantation from the same parental donor. For example, in one of the studies, the authors achieved complete hematopoietic chimerism and normal renal function without the need for immunosuppressive therapy 22–34 months after kidney transplantation [40]. In our centre, we did not perform bone marrow transplantation because there were no severe forms of immunodeficiency in our patients.

Previously, in our centre, we used a similar study approach to characterise the clinical and genetic landscape of nephropathic cystinosis, another very rare hereditary disease, and our study revealed two major nucleotide variants causative of disease to be characteristic for particular ethnic groups that live predominantly in Russia [41]. Thus, it is plausible to assume that the genetic landscape as well as clinical features of SIOD might also have population-, ethnic-, and race-specific features. For example, podocytic infolding glomerulopathy (PIG), a rare type of glomerulopathy that has been recently reported in a patient with SIOD, is predominantly found in patients from East Asia [42]. Interestingly, in one of the largest single-centre SIOD study cohorts in the Czech Republic, variant *c.2542G>T* was detected in 60% of patients compared to 74% in our study [19]. Not only the frequency but also the spectrum of PSVs in *SMARCAL1* in SIOD patients vary in different populations [13]. This, in turn, highlights the importance of continuous study of genetic diversity of SIOD in different populations.

Finally, determining the impact (or its absence) of various therapeutic approaches commonly used for SIOD, such as steroid therapy and kidney transplantation, on SMARCAL1’s biological functions could help in developing optimal treatment strategies in the case of SIOD patients with residual SMARCAL1 activity or some retaining functions of this protein.

## 4. Materials and Methods

### 4.1. Patients

A retrospective study of 21 paediatric patients with SIOD was performed at the Nephrology Department of the National Research Center for Children’s Health. All patients upon admittance to the clinic underwent a comprehensive laboratory and instrumental examination, including a kidney function assessment by glomerular filtration rate calculated using the Schwartz formula [43].

### 4.2. Genetic Study

#### 4.2.1. Sequencing

Two patients were not included in the genotyping study for different ethical and technical reasons (parental refusal, DNA quality). For the rest of the cohort, targeted NGS sequencing of a custom-built panel of genes involved in the pathogenesis of kidney diseases, previously described [6], was performed. NGS libraries were prepared using a KAPA HyperPlus Kit, Roche, New York, NY, USA according to the manufacturer’s protocol. DNA fragmentation time was 15 min to achieve the average fragment length of 350 bp. Target enrichment was carried out using KAPA HyperCap hybridisation probes, Roche, New York, NY, USA. Sequencing was performed on the MiSeq platform, Illumina, San Diego, CA, USA with V2 chemistry (500 cycles, pair-end reads) or NextSeq 2000, Illumina, San Diego, CA, USA with P3 chemistry (300 cycles, pair-end reads). Bioinformatics analysis was carried out according to GATK (Genome Analysis Toolkit) Best Practices recommendations [44]. For some patients, the *SMARCAL1* gene was sequenced by Sanger sequencing.

#### 4.2.2. Microsatellite Analysis

Microsatellite markers were studied using amplified fragment length polymorphism analysis. All markers were selected using the Marshfield NCBI (National Center for biotechnology Information) genetic map. Amplification of DNA fragments was performed by end-point PCR; the products of amplification were separated by polyacrylamide gel electrophoresis, followed by staining with ethidium bromide and visualisation in ultraviolet light. As a reference group, DNA samples from 19 unrelated donors residing in the Russian Federation who do not carry the *c.2542G>T*, p.E848* variant were used.

### 4.3. Statistical Analysis

All children with the syndrome were included in the statistical analysis, including those without PSVs in *SMARCAL1*. Quantitative data in the groups were checked for normal distribution using the Shapiro–Wilk test. Quantitative indicators with a distribution other than normal were presented as the median and 1st and 3rd quartiles. The statistical analysis of allele frequencies was based on the chi-square test for 2*2 table of conjugacy with alleles of two groups: the associated allele and all other alleles. To assess the degree of linkage disequilibrium (LD), we used the formula δ = (P_D_ − P_N_)/(1 − P_N_), and the confidence interval for δ-analysis was calculated as previously described [45,46]. Kaplan–Meier curves were drawn to estimate the overall and renal survival. Statistical analysis was performed using IBM SPSS software version 26. R version 4.4.1 was used for survival analysis.

## Figures and Tables

**Table 1:** The characteristics of the cohort.

Characteristics	
Males, number	12/21; 57%
Preterm delivery	15/16 **; 94%
Small foetus	9/16; 56%
Height at birth, SDS ***	−1.49 (−2.11; −0.83 *)
Weight at birth, SDS	−2.03 (−2.18; −1.62 *)
Median age at diagnosis (months)	45 (35; 69 *)
Proteinuria at diagnosis, median (g/day/1.73m^2^)	3.04 (1.43; 7.42 *)
Height at diagnosis, SDS	−5.13 (−6.14; −3.46 *)
Median age of CKD *** 5 (months)	62 (51; 79 *)
Growth at last observation, SDS	−5.92 (−7.44; −4.73 *)
Stroke in anamnesis	10/20 **; 50%
5-year survival	89%

* Median (25th; 75th percentiles). ** Total number is the number of patients for whom clinical data are available. *** SDS—Standard deviation score (for example, Height SDS = (x − X)/SD, where x–height of the child, X—average height of a child of the same age and sex, SD—standard deviation of height for this age and sex). *** CKD—chronic kidney disease.

**Table 2:** The clinical features identified in SIOD patients.

PatientID	Sex	Age	Proteinuria	Short Stature	Osseous Dysplasia (Incl. Deformations)	Multiple Abnormalities	Leukopenia, Immuno-Deficiency	Acute Cerebrovascular Accident	Visual Impairment	Hyper Pigmentation	Development Delay	CAKUT	Anemia	Congenital Heart Defect	Micro- Cephaly	Congenital Lung Disease	Bronchial Asthma
1	M	92 m. (7 y. 8 m.)	+	+	+	*+*	+	+	+	+	−	−	+	−	−	−	−
2	M	114 m. (9 y. 6 m.)	*+*	*+*	+	*+*	+	+	+	+	−	+	−	−	−	−	−
3	F	84 m. (7 y. 4 m.)	+	+	+	+	−	+	+	−	+	+	−	−	−	−	−
4	F	80 m. (6 y. 8 m.)	+	+	+	+	+	+	−	+	−	−	−	−	−	−	−
5	M	38 m. (3 y. 2 m.)	+	+	+	*+*	+	−	+	+	+	+	−	−	+	−	−
6	M	73 m. (6 y. 1 m.)	*+*	*+*	+	*+*	−	−	−	+	−	+	−	−	−	−	−
7	F	41 m. (3 y. 5 m.)	*+*	*+*	+	*+*	+	+	−	+	−	−	−	−	−	−	−
8	F	89 m. (7 y. 5 m.)	*+*	*+*	+	*+*	+	−	−	−	−	−	+	−	−	−	−
9	M	65 m. (5 y. 5 m.)	*+*	*+*	+	*+*	−	+	−	−	+	−	−	−	−	−	−
10	M	71 m. (5 y. 11 m.)	*+*	*+*	+	+	+	−	+	−	+	+	−	+	−	−	−
11	M	98 m. (8 y. 2 m.)	*+*	*+*	+	*+*	−	−	−	−	−	−	−	−	−	−	+
12	F	62 m. (5 y. 2 m.)	*+*	*+*	+	*+*	+	−	+	−	−	+	−	−	−	−	−
13	M	59 m. (4 y. 11 m.)	*+*	*+*	+	*+*	+	−	+	−	−	−	−	−	−	−	−
14	F	40 m. (3 y. 4 m.)	*+*	*+*	+	*+*	+	+	+	+	+	−	−	+	−	+	−
15	F	15 m. (1 y. 3 m.)	*+*	*+*	+	*+*	+	−	−	−	−	−	−	−	−	−	−
16	F	9 m.	*+*	*+*	−	−	+	−	+	−	+	+	−	−	−	−	−
17	M	44 m. (3 y. 8 m.)	*+*	*+*	+	*−*	+	−	−	+	−	−	−	+	−	−	−
18	M	101 m. (8 y. 5 m.)	*+*	*+*	+	+	+	−	−	+	+	−	−	−	−	−	−
19	M	106 m. (8 y. 10 m.)	*+*	*+*	+	+	−	+	−	−	−	−	−	−	−	−	−
20	F	46 m. (3 y. 10 m.)	*+*	*+*	+	−	+	+	−	−	+	−	−	−	−	−	−
21	M	99 m. (8 y. 3 m.)	*+*	*+*	+	−	+	+	+	−	−	−	+	−	−	−	−

**Table 3:** The causative PSVs in *SMARCAL1* identified in SIOD patients.

Patient ID	Sex	Age	Allele 1	Reference, ACMG Classification, (Frequency, gnomAD v.4.1.0)	Allele 2	Reference, ACMG Classification, (Frequency, gnomAD v.4.1.0)	
1	M	92 m. (7 y. 8 m.)	*c.2542G>T*, p.E848*	[8], Pathogenic (0.008%)	*c.355_500del*, p.L119Gfs*22	Pathogenic (n/a)	Deceased
2	M	114 m. (9 y. 6 m.)	*c.2542G>T*, p.E848*	[8], Pathogenic (0.008%)	*c.1710+5G>A*	[6], Likely pathogenic (n/a)	Deceased
3	F	84 m. (7 y. 4 m.)	*c.2542G>T*, p.E848*	[8], Pathogenic (0.008%)	*c.2290C>T*, p.R784W	[8], Pathogenic (0.008%)	Deceased
4	F	80 m. (6 y. 8 m.)	*c.2459G>A*, p.R820H	[8], Pathogenic (0.007%)	*c.2562del*, p.E855Kfs*12	Pathogenic (n/a)	Deceased
5	M	38 m. (3 y. 2 m.)	*c.2542G>T*, p.E848*	[8], Pathogenic (0.008%)	*c.2542G>T*, p.E848*	[8], Pathogenic (0.008%)	Deceased
6	M	73 m. (6 y. 1 m.)	*c.2542G>T*, p.E848*	[8], Pathogenic (0.008%)	*c.2533_2534del*, p.L845Dfs*6	Pathogenic (n/a)	Deceased
7	F	41 m. (3 y. 5 m.)	*c.2542G>T*, p.E848*	[8], Pathogenic (0.008%)	*c.2542G>T*, p.E848*	[8], Pathogenic (0.008%)	Alive
8	F	89 m. (7 y. 5 m.)	*c.2542G>T*, p.E848*	[8], Pathogenic (0.008%)	*c.1582A>C*, p.S528R	Likely pathogenic (n/a)	Deceased
9	M	65 m. (5 y. 5 m.)	*c.2542G>T*, p.E848*	[8], Pathogenic (0.008%)	*c.2542G>T*, p.E848*	[8], Pathogenic (0.008%)	Deceased
10	M	71 m. (5 y. 11 m.)	*c.415_416del*, p.L139Efs*2	[8], Pathogenic (0.004%)	*c.1933C>T*, p.R645C	[8], Pathogenic (0.004%)	N/A
11	M	98 m. (8 y. 2 m.)	*c.2542G>T*, p.E848*	[8], Pathogenic (0.008%)	*c.1010T>C*, p.L337P	Likely pathogenic (n/a)	N/A
12	F	62 m. (5 y. 2 m.)	*c.2542G>T*, p.E848*	[8], Pathogenic (0.008%)	*c.1736C>T*, p.S579L	[8], (0.004%)	N/A
13	M	59 m. (4 y. 11 m.)	*c.994del*, p.R332Dfs*49	Pathogenic (n/a)	*c.2070dup*, p.K691*	Pathogenic (n/a)	N/A
14	F	40 m. (3 y. 4 m.)	*c.2542G>T*, p.E848*	[8], Pathogenic (0.008%)	*c.2551A>T*, p.K851*	Pathogenic (n/a)	Deceased
15	F	15 m. (1 y. 3 m.)	*c.2542G>T*, p.E848*	[8], Pathogenic (0.008%)	*c.2149_2150dup*, p.L718Sfs*13	[8] Pathogenic (0.0003%)	Alive
16	F	9 m.	*c.2542G>T*, p.E848*	[8], Pathogenic (0.008%)	*c.2542G>T*, p.E848*	[8], Pathogenic (0.008%)	N/A
17	M	44 m (3 y. 8 m.)	*c.939delC*, pT314fs	_________	*c.1451T>A*, p.V484E	_________	Alive
18	M	101 m. (8 y. 5 m.)	*c.2542G>T*, p.E848*	[8], Pathogenic (0.008%)	-		Deceased
19	M	106 m. (8 y. 10 m.)	*c.2459G>A*, p.R820H	[8], Pathogenic (0.007%)	-		Deceased

**Table 4:** Microsatellite analysis of SIOD patients.

Marker	D2S1345	D2S2382	*c.2542G>T*, p.E848*	D2S163
Coordinate (cM)	210.43	213.49	213.49	218.45
Coordinate (kB)	215.165	217.048	217.34	220.5
Patient № 5	3	1	T	5
	1	1	T	5
Patient № 7	2	1	T	7
	1	1	T	5
Patient № 16	3	4	T	7
	1	1	T	6
Patient № 1	4	1	T	7
	3	1	T	6
Patient № 3	6	12	T/G	10
	4	1		6
Patient № 2	4	12	T/G	8
	3	1		6
Patient № 6	6	5	T/G	6
	3	1		5

**Table 5:** Microsatellite marker analysis.

Marker	Coordinate, cM	Allele	*p*-Value	δ ± 95 CI
D2S1345	210.43	1	0.005	0.472 ± 0.137
D2S2382	213.49	1	0.015	0.804 ± 0.135
*c.2542G>T*, p.E848*	213.49	t	-----	--------
D2S163	218.45	5	0.015	0.419 ± 0.156

## Data Availability

Data are available only on reasonable request from the corresponding author due to patients’ privacy/ethical restrictions.

## Abbreviations

Angptl3	angiopoietin-like 3
AH	arterial hypertension
CAKUT	congenital anomalies of the kidney and urinary Tract
CKD	chronic kidney disease
DNA	deoxyribonucleic acid
ESRD	end-stage renal disease
FD	Federal District
GATK	Genome Analysis Toolkit
HARP	HepA-related protein
LD	linkage disequilibrium
mRNA	messenger ribonucleic acid
NCBI	National Center for biotechnology Information
NGS	next generation sequencing
NS	nephrotic syndrome
OMIM	online Mendelian inheritance in man
OS	overall survival rate
PCR	polymerase chain reaction
PIG	podocytic infolding glomerulopathy
RRT	renal replacement therapy
SDS	standard deviation score
SIOD	Schimke immuno-osseous dysplasia
SNF2	sucrose non-fermenting type 2
SMARCAL1	SWI/SNF-related matrix-associated actin-dependent regulator of chromatin subfamily A-like protein
*SMARCAL1*	gene encoding SMARCAL1 protein
SRNS	steroid-resistant nephrotic syndrome
